# Transcranial focused ultrasound to the posterior cingulate cortex modulates default mode network and subjective experience: an fMRI pilot study

**DOI:** 10.3389/fnhum.2024.1392199

**Published:** 2024-06-04

**Authors:** Brian Lord, Joseph L. Sanguinetti, Lisannette Ruiz, Vladimir Miskovic, Joel Segre, Shinzen Young, Maria E. Fini, John J. B. Allen

**Affiliations:** ^1^SEMA Lab, Psychology Department, Center for Consciousness Studies, University of Arizona, Tucson, AZ, United States; ^2^Sanmai Technologies, PBC, Sunnyvale, CA, United States; ^3^X, the Moonshot Factory, Mountain View, CA, United States

**Keywords:** transcranial focused ultrasound, neuromodulation, non-invasive brain stimulation, default mode network, mindfulness, fMRI

## Abstract

**Background:**

Transcranial focused ultrasound (TFUS) is an emerging neuromodulation tool for temporarily altering brain activity and probing network functioning. The effects of TFUS on the default mode network (DMN) are unknown.

**Objective:**

The study examined the effects of transcranial focused ultrasound (TFUS) on the functional connectivity of the default mode network (DMN), specifically by targeting the posterior cingulate cortex (PCC). Additionally, we investigated the subjective effects of TFUS on mood, mindfulness, and self-related processing.

**Methods:**

The study employed a randomized, single-blind design involving 30 healthy subjects. Participants were randomly assigned to either the active TFUS group or the sham TFUS group. Resting-state functional magnetic resonance imaging (rs-fMRI) scans were conducted before and after the TFUS application. To measure subjective effects, the Toronto Mindfulness Scale, the Visual Analog Mood Scale, and the Amsterdam Resting State Questionnaire were administered at baseline and 30 min after sonication. The Self Scale and an unstructured interview were also administered 30 min after sonication.

**Results:**

The active TFUS group exhibited significant reductions in functional connectivity along the midline of the DMN, while the sham TFUS group showed no changes. The active TFUS group demonstrated increased state mindfulness, reduced Global Vigor, and temporary alterations in the sense of ego, sense of time, and recollection of memories. The sham TFUS group showed an increase in state mindfulness, too, with no other subjective effects.

**Conclusions:**

TFUS targeted at the PCC can alter DMN connectivity and cause changes in subjective experience. These findings support the potential of TFUS to serve both as a research tool and as a potential therapeutic intervention.

## 1 Introduction

The default mode network (DMN) has been the subject of extensive research since it was initially defined by Raichle et al. (2001). The DMN is anchored by two midline nodes at the medial prefrontal cortex (mPFC) and the posterior cingulate cortex (PCC) with adjacent bilateral nodes in the angular gyri and middle temporal gyri (Andrews-Hanna et al., 2010). While theoretical ideas and debate about the function of the DMN continue to evolve (Andrews-Hanna et al., 2014; Yeshurun et al., 2021), it appears to play an essential role in the inner processes of mind-wandering, planning, and self-related processing (Raichle, 2015).

The DMN has been implicated in several disorders, including depression (Scalabrini et al., 2020; Zhou et al., 2020; Ju et al., 2022), addiction (Zhang and Volkow, 2019), autism (Padmanabhan et al., 2017), ADHD (Posner et al., 2014; Harikumar et al., 2021), and schizophrenia (Hu et al., 2017). Collectively, the clinical literature suggests that a breakdown in the regulation of the DMN may be one mechanism underlying these disorders (e.g., abnormal DMN activity leading to increased rumination in depression) (Lydon-Staley et al., 2019; Zhou et al., 2023). Thus, potential treatments that normalize DMN function may be called for (Holtzheimer and Mayberg, 2011; Scalabrini et al., 2020).

Empirical research with psychedelics has implicated the DMN in constructing varying states of consciousness and representations of the self, especially the “narrative self,” in which the self is the object of thought (Nour and Carhart-Harris, 2017). The current scientific consensus establishes that disruption of resting state functional connectivity within the DMN is a central mechanism that drives their profound psychological and therapeutic effects (Carhart-Harris et al., 2012; Gattuso et al., 2022). Carhart-Harris et al. (2012) found that the degree to which psilocybin decreased cerebral blood flow and connectivity of the mPFC and PCC predicted the magnitude of the self-altering effects.

The practice of meditation can also alter resting-state brain activity, including the DMN. Brewer et al. (2013) found reduced DMN connectivity in experienced meditators during meditation in the MRI scanner. The same group demonstrated that meditators could volitionally reduce activity in their PCC using real-time fMRI neurofeedback, and that this reduction correlated with their internal meditative experience (Garrison et al., 2013).

Neurophenomenological analysis suggested that PCC deactivation was associated with an experience of “undistracted awareness” and “effortless doing” (Garrison et al., 2013). This led Brewer and colleagues (Brewer et al., 2013; Brewer and Garrison, 2014) to suggest that PCC activity plays a role in self-referential processing, particularly a tendency to “get caught up in” one’s experience. In the mindfulness literature, this quality may also be described as a lack of equanimity (Desbordes et al., 2015). Importantly, mindfulness training, as demonstrated by Korponay et al. (2019), does not simply reduce DMN activity but rather enhances one’s ability to control and inhibit it when necessary.

The extant fMRI literature in healthy individuals, clinical studies, psychedelics, and contemplative experiments all converge on the major role of the DMN in internal thought and self-related processing (Andrews-Hanna, 2012; Andrews-Hanna et al., 2014; Davey et al., 2016; Nour and Carhart-Harris, 2017; Carhart-Harris and Friston, 2019; Davey and Harrison, 2022). Non-invasive brain stimulation techniques that enable targeted manipulation of DMN regions would offer an opportunity to estimate the causal relationship between DMN activity and internal processes, paving the way to potential effective therapeutics.

A promising method for non-invasive brain modulation is transcranial focused ultrasound (TFUS). TFUS modulates brain regions with pulsed beams of focused ultrasound with millimeter precision (Blackmore et al., 2019). Unlike other non-invasive brain stimulation techniques like transcranial electrical stimulation (TES) or transcranial magnetic stimulation (TMS), TFUS can effectively reach deep subcortical regions like the thalamus (Legon et al., 2018; Kim et al., 2023) by adjusting the focal depth of the ultrasound beam. The safety profile of TFUS is favorable: the current picture is that adverse events only occur when stimulation is too long and/or intense, far in excess of FDA safety limits, causing thermal and/or biophysical damage to the targeted tissue or unintentional opening of the blood–brain barrier (Pasquinelli et al., 2019).

TFUS acts through a combination of potential mechanisms, including thermal, mechanical, and cavitation effects as a result of the acoustic energy interacting with neural tissue (Dell’Italia et al., 2022). Reviews suggest that it can be both excitatory and inhibitory in its effects (Zhang et al., 2021), but there is not always a clear differentiation between the two, as some studies show both excitatory and inhibitory effects simultaneously (Verhagen et al., 2019; Yoon et al., 2019), while others show state-dependent (Yang et al., 2021) or cell type-dependent (Wattiez et al., 2017) responses. Given that similar effects can arise from bidirectional mechanisms (e.g., inhibition of excitatory neurons or stimulation of inhibitory neurons can both produce suppression), it is more accurate to describe the neuromodulatory effects at the tissue-level. Network (Folloni et al., 2019) and distal (Cain et al., 2021) effects can also manifest, as can delayed, offline effects (Carhart-Harris et al., 2012). Sanguinetti et al. (2020) found that targeting the right prefrontal cortex induced mood enhancement and decreased functional connectivity in regions distal from the target.

Given these capabilities, TFUS is an ideal candidate for non-invasively modulating the DMN. The aim of this pilot study was to target the PCC using TFUS parameters that are expected to suppress neural firing (Dell’Italia et al., 2022), specifically by utilizing a low duty cycle value of 5.26%. The hypothesis was that this approach would enable modulation of the resting state connectivity from that node to the rest of the network. We also hypothesized that this would induce changes in phenomenology that relate to DMN activity, specifically mindfulness and self-referential processing. As a proof-of-concept, this would pave the way for TFUS to serve as a tool to probe network functioning and be used as a therapeutic intervention.

## 2 Materials and methods

### 2.1 Subjects

Thirty healthy subjects (18 female, average age 19.8 years) participated in this study. Exclusion criteria were: use of tobacco/nicotine, history of head injury, uncorrected hearing or vision impairment, history of brain or mental illness (including drug and/or alcohol dependence), use of pharmaceuticals (including psychotropic drugs), sleep disorders, drug or alcohol intoxication, history of epilepsy, history of migraines, metal implants in their head, and history of cardiac problems. Inclusion criteria were: age 18–77, normal or corrected vision, and proficient enough in English to read the consent form. Subjects received either actual or sham TFUS in a single-blind, between-subjects design.

### 2.2 Procedure

**Figure 1:**
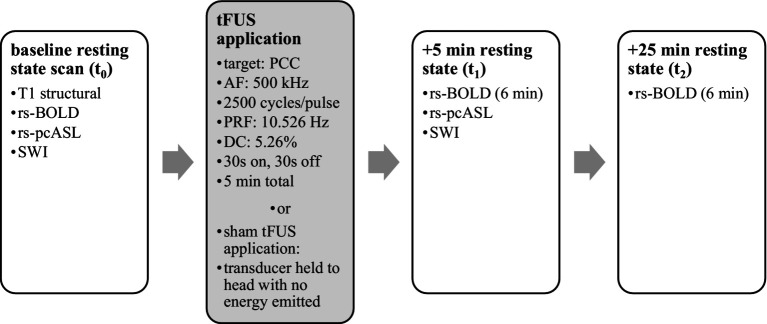
Timeline of MRI data acquisition and TFUS application. Data were gathered before and after TFUS application.

After screening and consent, subjects were given subjective rating scales. Subsequently, they underwent four MRI scans: T1 structural, baseline functional resting state (rs-BOLD), arterial spin labeling (rs-pcASL), and susceptibility weighted imaging (SWI). During functional scans, subjects were instructed to stare at a fixation cross and allow their thoughts to flow naturally. Subjects were removed from the scanner, and real-time neuronavigation (Visor2, ANT Neuro, Netherlands) was used to apply either active or sham TFUS to their ventral PCC based on their individual structural MRI. Sham TFUS was performed by holding an unplugged transducer against their head. Subjects then returned to the MRI scanner for further measurements. Functional MRI scans were captured in the 5 min immediately after application (t_1_) and at 25 min after application (t_2_) (see Figure 1). Post-sonication SWI and pcASL scans were also taken after t_1_. Upon exiting the scanner, final subjective rating scales were taken, and the subject was debriefed.

### 2.3 Subjective ratings

Before any MRI scans, subjects responded to the Visual Analog Mood Scale (VAMS) (Luria, 1975) and the Toronto Mindfulness Scale (TMS) (Lau et al., 2006). After TFUS and all subsequent MRI scans, they responded to the same scales again, along with the Self Scale (Lebedev et al., 2015) and the Amsterdam Resting-State Questionnaire (ARSQ) (Diaz et al., 2013).

### 2.4 Post experiment questions

Structured post-experiment questions were asked of each participant. Participants were asked to guess if they were in the “stimulation or placebo condition,” whether the ultrasound changed their “overall mental state,” if they heard anything from the transducer, and if they had changes to their “inner talk- or thinking-space.”

### 2.5 MRI scans

Functional BOLD images were acquired on a Siemens Skyra 3-Tesla scanner using EPI gradient echo sequence (TR = 1800 ms; TE = 25 ms; flip angle = 90; FOV = 192 mm; acquisition voxel size 3 mm × 3 mm × 3 mm). T1-weighted anatomical images were also acquired for neuronavigation and registration of the functional scans (MP-RAGE; TR = 2,500 ms; TE = 4.35 ms; TI = 900 ms; flip angle = 8; FOV = 256 mm).

### 2.6 Acoustic intensity measurements

**Figure 2:**
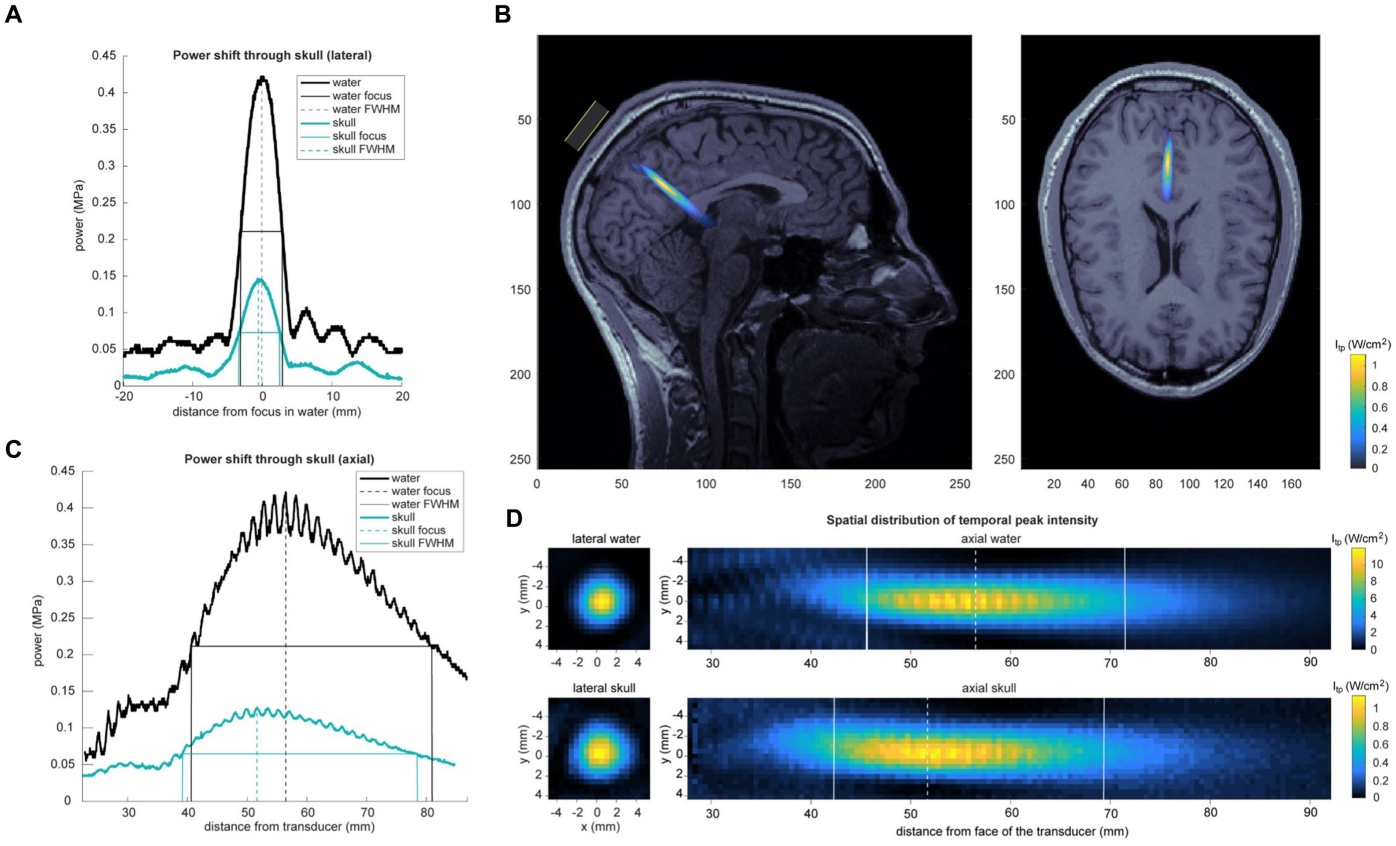
Acoustic intensity measurements. **(A)** Skull attenuation and geometric deformation of acoustic temporal peak pressure recorded in water and through a sample of cadaver parietal bone (“skull”). Center of the beam and FWHM are displayed. Lateral shift displayed in **(A)** and axial shift in **(C)**. The periodic variation in peak intensity in the axial plane in panel C is thought to be due to standing waves created by the annular geometry of the transducer. **(B)** Skull-attenuated ultrasound intensity map is overlaid on a single subject’s MRI. Estimation of peak focus was determined using recorded neuronavigation coordinates from that subject’s TFUS session. **(D)** Spatial distribution of temporal peak intensity of the ultrasound beam in water (top) and through a cadaver parietal skull piece (bottom). Left panel shows lateral spatial topography of temporal peak intensity of the beam at the axial peak; right panel displays axial topography.

Acoustic intensity was measured using a custom-built water tank setup. Data were recorded using a needle hydrophone (HNR-0500; Onda, Sunnyvale, CA, United States) with a geometric diameter of 2.5 mm. A scan volume of 12 mm (x), 12 mm (y), 68 mm (axial) was collected using 0.508 mm steps in degassed water. Pressure and intensity were calculated from the voltage recordings from the hydrophone. MI was calculated with a derated peak pressure using the attenuation coefficient of soft tissue (0.3 dB/cm) (Abbott, 1999). The measured output of this wave in degassed free water shows a peak negative pressure of 0.422 MPa. The output of the beam through a hydrated sample of cadaver parietal bone showed a peak negative pressure of 0.130 MPa (a 69.3% decrease). See Figure 2. Full properties of the wave output are presented in Table 1. The skull caused little deviation of the focus in the lateral plane, but in the axial plane focus became more shallow (axial focus water = 56.6 mm; skull = 51.7 mm; difference = −4.8 mm). See Table 2. The periodic variation in peak intensity in the axial plane (panel C of Figure 2) is thought to be due to standing waves created by the annular geometry of the transducer.

**Table 1:** Acoustic wave analysis.

Measurement	Water	Skull	Units	% decrease through skull	Description
I_SPTP_	11.91	1.13	W/cm^2^	90.5	Spatial peak temporal peak intensity
I_SPPA_	5.58	0.46	W/cm^2^	91.8	Spatial peak pulse average intensity
I_SPTA_	293.37	23.98	mW/cm^2^	91.8	Spatial peak temporal average intensity
P_SPTP_	421.57	129.60	kPa	69.3	Spatial peak temporal peak pressure
P_SPPA_	260.52	73.86	kPa	71.7	Spatial peak pulse average pressure
P_SPTA_	13.70	3.88	kPa	71.6	Spatial peak temporal average pressure
MI^a^	0.60	0.18	–	69.3	Mechanical index

a MI derated for soft tissue from water only.

**Table 2:** Skull distortion measurement.

	Axial			Lateral		
Measurement	Water	Skull	Shift through skull	Water	Skull	Shift through skull
Focal peak	56.5 mm	51.7 mm	−4.8 mm	0.0 mm	−0.5 mm	−0.5 mm
Intensity FWHM start	45.6 mm	42.3 mm	−3.3 mm	−2.3 mm	−2.4 mm	−0.1 mm
Intensity FWHM end	71.4 mm	69.4 mm	−2.1 mm	2.2 mm	1.8 mm	−0.5 mm
Intensity FWHM length	25.9 mm	27.1 mm	1.2 mm	4.5 mm	4.2 mm	−0.4 mm
Power FWHM start	40.6 mm	30.2 mm	−10.5 mm	−3.1 mm	−3.3 mm	−0.3 mm
Power FWHM end	81.9 mm	78.4 mm	−3.5 mm	2.9 mm	2.5 mm	−0.4 mm
Power FWHM length	41.3 mm	48.3 mm	7.0 mm	6.0 mm	5.8 mm	−0.1 mm

### 2.7 TFUS stimulation

Subjects were seated comfortably. An MRI-guided stereotactic system (Visor2, ANT Neuro, Netherlands) was used to guide TFUS targeting to the participant’s PCC. The focused ultrasound was delivered by a custom 4-channel ring transducer (Sonic Concepts, Bothell, WA, United States) with an outer diameter of 64 mm that uses a sealed membrane filled with degassed water for coupling, which is then housed inside a custom 3D-printed casing. The transducer was driven by an acoustic amplifier (TPO-203, Sonic Concepts, Bothell, WA, USA), with the ultrasound beam having the following parameters: acoustic frequency (AF) = 500 kHz, pulse repetition frequency (PRF) = 10.526 Hz, pulse repetition period (PRP) = 95 ms, pulse duration (PD) = 5 ms or 2,500 cycles, duty cycle = 5.26%. Badran and colleagues used similar parameters to suppress the pain pathway, except they used a 650 kHz acoustic frequency (Badran et al., 2020; Li et al., 2021).

The beam was focused to fixed distance of 55 mm, corresponding to the average distance of the PCC from the surface of the scalp where the transducer is applied. The target was determined by inspection of each subject’s anatomical scan. The ventral PCC was chosen due to its greater association with internal directed thought rather than the cognitive control functions associated with the dorsal PCC (Leech and Sharp, 2014).

The subject’s head was registered to their structural MRI in the Visor2 neuronavigation software using three fiducials (nasion and ears) and their scalp surface. The transducer was held firmly against the subject’s head, using individual MR-guided neuronavigation, with gel applied to the scalp, to deliver 30-s stimulus intervals followed by 30-s rest periods. The pattern of [30s ON, 30s OFF] was repeated five times consecutively, totaling a 5-min duration. Participants were instructed to sit quietly with their eyes open. The researcher periodically (about every 1.5 min) asked them how they were doing, if they felt anything unusual, and if they would like to continue.

### 2.8 MRI analysis

Results included in this manuscript come from analyses performed using CONN (Whitfield-Gabrieli and Nieto-Castanon, 2012) (RRID:SCR_009550) release 21.a (Nieto-Castanon and Whitfield-Gabrieli, 2021) and SPM (Penny et al., 2011) (RRID:SCR_007037) release 12.7771.

#### 2.8.1 Preprocessing

Functional and anatomical data were preprocessed using a flexible preprocessing pipeline (Nieto-Castanon, 2020a) including realignment with correction of susceptibility distortion interactions, slice timing correction, outlier detection, direct segmentation and MNI-space normalization, and smoothing. Functional data were realigned using SPM realign & unwarp procedure (Andersson et al., 2001), where all scans were coregistered to a reference image (first scan of the first session) using a least squares approach and a 6 parameter (rigid body) transformation (Friston et al., 1995), and resampled using b-spline interpolation to correct for motion and magnetic susceptibility interactions. Temporal misalignment between different slices of the functional data (acquired in interleaved Siemens order) was corrected following SPM slice-timing correction (STC) procedure (Henson et al., 1999; Sladky et al., 2011), using sinc temporal interpolation to resample each slice BOLD timeseries to a common mid-acquisition time. Potential outlier scans were identified using ART (Whitfield-Gabrieli et al., 2011) as acquisitions with framewise displacement above 0.9 mm or global BOLD signal changes above 5 standard deviations (Nieto-Castanon, 2022; Power et al., 2014), and a reference BOLD image was computed for each subject by averaging all scans excluding outliers. Functional and anatomical data were normalized into standard MNI space, segmented into grey matter, white matter, and CSF tissue classes, and resampled to 2 mm isotropic voxels following a direct normalization procedure (Calhoun et al., 2017; Nieto-Castanon, 2022) using SPM unified segmentation and normalization algorithm (Ashburner and Friston, 2005; Ashburner, 2007) with the default IXI-549 tissue probability map template. Last, functional data were smoothed using spatial convolution with a Gaussian kernel of 8 mm full width half maximum (FWHM).

#### 2.8.2 Denoising

In addition, functional data were denoised using a standard denoising pipeline (Nieto-Castanon, 2020b) including the regression of potential confounding effects characterized by white matter timeseries (5 CompCor noise components), CSF timeseries (5 CompCor noise components), motion parameters and their first order derivatives (12 factors) (Friston et al., 1996), outlier scans (below 35 factors) (Power et al., 2014), session and task effects and their first order derivatives (6 factors), and linear trends (2 factors) within each functional run, followed by bandpass frequency filtering of the BOLD timeseries (Hallquist et al., 2013) between 0.008 Hz and 0.09 Hz. CompCor (Behzadi et al., 2007; Chai et al., 2012) noise components within white matter and CSF were estimated by computing the average BOLD signal as well as the largest principal components orthogonal to the BOLD average, motion parameters, and outlier scans within each subject’s eroded segmentation masks. From the number of noise terms included in this denoising strategy, the effective degrees of freedom of the BOLD signal after denoising were estimated to range from 130.9 to 147.6 (average 144.5) across all subjects (Nieto-Castanon, 2022).

#### 2.8.3 First-level analysis

ROI-to-ROI connectivity (RRC) matrices were estimated characterizing the functional connectivity between each pair of regions among 100 ROIs (Schaefer et al., 2018). Functional connectivity strength was represented by Fisher-transformed bivariate correlation coefficients from a general linear model (weighted-GLM) (Nieto-Castanon, 2020c), estimated separately for each pair of ROIs, characterizing the association between their BOLD signal timeseries. Individual scans were weighted by a boxcar signal characterizing each individual task or experimental condition convolved with an SPM canonical hemodynamic response function and rectified.

#### 2.8.4 Group-level analyses

Group-level analyses were performed using a General Linear Model (GLM) (Nieto-Castanon, 2020d). A contrast that averages both post-stimulation conditions against the baseline (t_1_ + t_2_ > baseline) was designed to estimate a general model of the effects of tFUS for each active and sham group. Time points (t_1_ and t_2_) were combined to enhance the statistical power of the model due to the small sample size. Individual time point models were also estimated for each condition (t_1_ > baseline and t_2_ > baseline) in an exploratory secondary analysis to examine the temporal nature of the effects. For each individual connection a separate GLM was estimated, with first-level connectivity measures at this connection as dependent variables (one independent sample per subject and one measurement per task or experimental condition, if applicable), and groups or other subject-level identifiers as independent variables. Connection-level hypotheses were evaluated using multivariate parametric statistics with random-effects across subjects and sample covariance estimation across multiple measurements. Inferences were performed at the level of individual clusters (groups of contiguous connections). Cluster-level inferences were based on nonparametric statistics using Threshold Free Cluster Enhancement (TFCE) (Smith and Nichols, 2009), with 1,000 residual-randomization iterations, and ROIs sorted using optimal leaf ordering based on ROI-to-ROI anatomical proximity and functional similarity metrics (Bar-Joseph et al., 2001; Nieto-Castanon, 2020e). For the primary effects model (t_1_ + t_2_ > baseline), results were conservatively thresholded using a combination of a cluster-forming *p* < 0.001 connection-level threshold and a familywise corrected p-FDR < 0.01 cluster-mass threshold. For the secondary exploratory analysis of individual time points, less conservative thresholds were used (connection-level *p* < 0.05 and cluster-level *p* < 0.05).

## 3 Results

### 3.1 Functional connectivity

**Figure 3:**
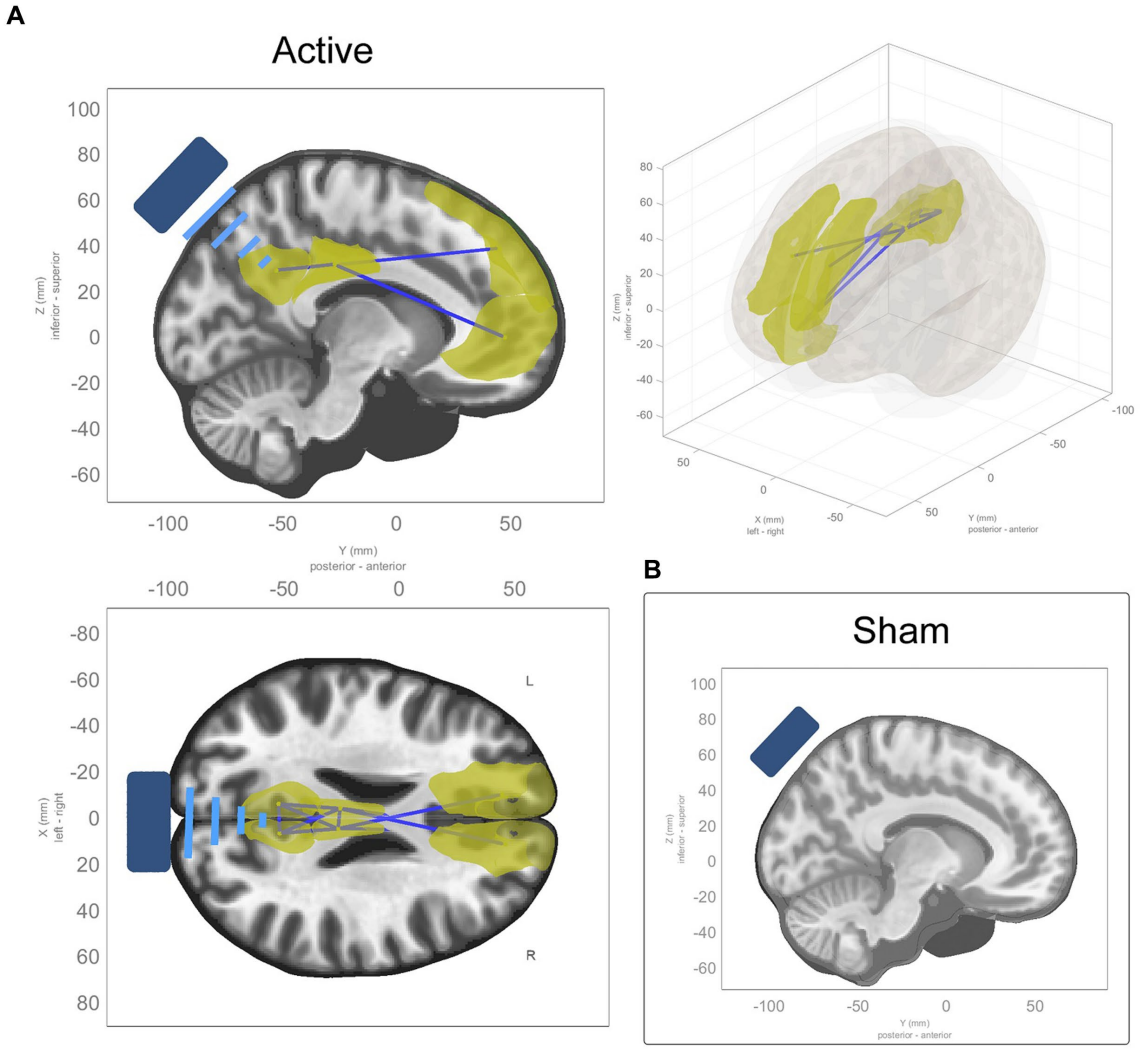
ROI-to-ROI functional connectivity changes from baseline to average of t_1_ and t_2_. **(A)** Sagittal, coronal, and axial views of significant ROI-to-ROI functional connectivity changes in active TFUS condition, all of which were decreases in functional connectivity (represented by blue connecting lines, with the affected ROIs highlighted in yellow) within and along the midline of the DMN and cingulate cortex. **(B)** No significant ROI-to-ROI functional connectivity changes found in the sham TFUS condition.

ROI-to-ROI analysis revealed significant decreases in connectivity in the active group within 1 cluster comprising 8 ROIs and 11 connections between them, while there were no significant changes in the sham group (see Table 3). The cluster comprised a decrease in connectivity between midline nodes of the DMN. There were reductions in connectivity across hemispheres in the cingulate cortex, and the medial and dorsolateral prefrontal cortex reduced in connectivity with the posterior cingulate (see Figure 3). The same model was estimated for the sham group with no significant effects found. While a single model that contrasted active and sham groups found no significant effects, the effects found in the active model were highly significant after conservative corrections were made.

**Table 3:** ROI-to-ROI functional connectivity analysis of effects of TFUS.

Analysis unit	Statistic	p-unc	p-FDR	p-FWE
Cluster 1	Score = 209.67	0.0008	0.0237	0.0050
	Mass = 576.39	0.0001	0.0048	0.0010
	Size = 22	0.0001	0.0038	0.0010
Right Cingulate Posterior – Left Cingulate Posterior	T(14) = −7.20	<0.0001	0.0159	
Left Cingulate Posterior – Right Precuneus Posterior Cingulate	T(14) = −5.67	<0.0001	0.0507	
Left Cingulate Posterior – Right Medial Prefrontal Cortex	T(14) = −5.53	0.0001	0.0507	
Right Cingulate Posterior – Right Medial Prefrontal Cortex	T(14) = −5.18	0.0001	0.0555	
Left Cingulate Posterior – Right Dorsal Prefrontal Cortex	T(14) = −5.05	0.0002	0.0604	
Right Cingulate Posterior – Right Precuneus Posterior Cingulate	T(14) = −4.79	0.0003	0.0808	
Right Cingulate Posterior – Left Precuneus Posterior Cingulate	T(14) = −4.71	0.0003	0.0882	
Left Cingulate Posterior – Left Precuneus Posterior Cingulate	T(14) = −4.51	0.0005	0.0989	
Right Precuneus Posterior Cingulate – Left Precuneus Posterior Cingulate	T(14) = −4.40	0.0006	0.0989	
Right Cingulate Posterior – Left Dorsal Prefrontal Cortex	T(14) = −4.35	0.0007	0.0989	
Left Cingulate Posterior – Left Medial Prefrontal Cortex	T(14) = −4.19	0.0009	0.1084	

p-unc, uncorrected *p*-value; p-FDR, false discovery rate corrected *p*-value; p-FWE, family wise error rate corrected *p*-value.

**Figure 4:**
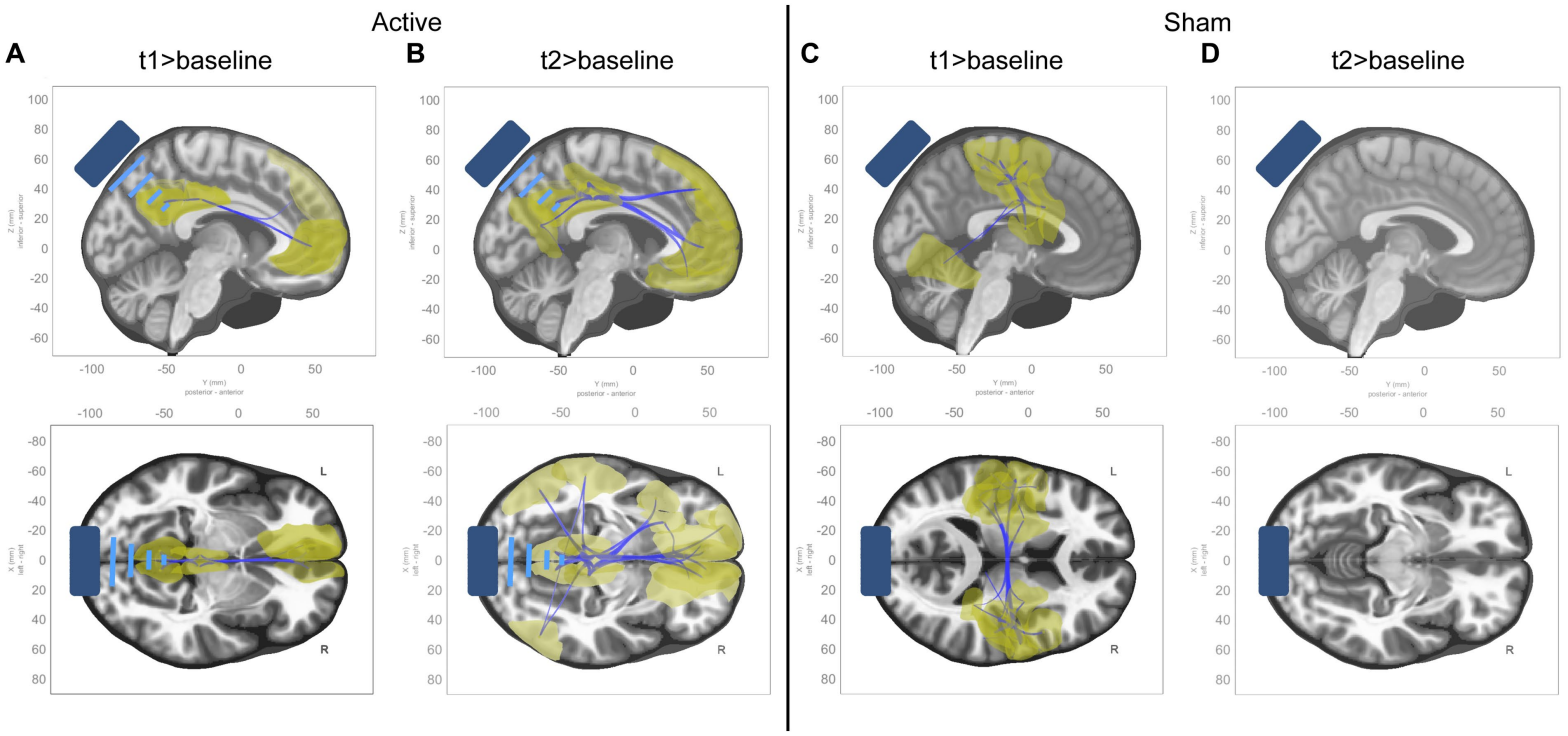
ROI-to-ROI functional connectivity changes from baseline to t_1_ and t_2_ in active condition. Sagittal and axial views of significant ROI-to-ROI functional connectivity changes in each timepoint compared to baseline for each condition, all of which were decreases in functional connectivity (represented by blue connecting lines, with the affected ROIs highlighted in yellow). **(A)** Contrast of t_1_ to baseline in active condition. **(B)** Contrast of t_2_ to baseline in active condition. **(C)** Contrast of t_1_ to baseline in sham condition. **(D)** Contrast of t_2_ to baseline in sham condition.

Models were estimated for the effects at individual time points (t_1_ > baseline and t_2_ > baseline) within each condition. In t_1_ of the active condition, reductions in connectivity were seen along the midline of the DMN within 1 cluster comprising 7 ROIs and 11 connections between them (see Supplementary Table S1 for details). In t_2_, these effects are more diffuse, extending to the left and right inferior parietal lobes, the left and right medial parietal lobes, and the left temporal pole, within 1 cluster comprising 17 ROIs and 45 connections between them (see Supplementary Table S2 for details). See panels A and B in Figure 4.

In the sham condition, connectivity reductions were seen in t_1_ primarily within somatomotor and dorsal attention networks, within 1 cluster comprising 26 ROIs and 69 connections between them (see Supplementary Table S3 for details). No effects were seen in t_2_. See panels C and D in Figure 4.

### 3.2 Subjective ratings

#### 3.2.1 Psychometric scales

Due to small sample sizes (*n* = 15 for each condition) and skewed results in many test questions, we opted for non-parametric tests (Wilcoxon rank sum tests and linear mixed models) instead of parametric tests to analyze the psychometric scales. The Wilcoxon rank-sum test compares ranks instead of raw data, and linear mixed models are less sensitive to violations of normality. These tests are more robust, less affected by outliers or skewed data, reducing the risk of drawing incorrect conclusions.

##### 3.2.1.1 Toronto Mindfulness Scale

We estimated a linear mixed model that included session (pre and post) and condition (active and sham) as fixed effects, their interaction, and a random intercept for subjects, for the Toronto Mindfulness Scale score. The formula for the model is Toronto Mindfulness Scale Score ~ Session * Condition + (1 | Subject) (see Table 4). There was a significant main effect of session (*p* = 0.0001), with the post session having higher values than the pre session. However, the main effect of condition (*p* = 0.552) and the interaction between session and condition (*p* = 0.173) were not significant.

**Table 4:** Effects estimates for the linear mixed model for Toronto Mindfulness Scale.

Fixed effect	Estimate	Std. Error	df	*t* value	*p*-value
(Intercept)	24.467	2.594	38.112	9.432	<0.001***
Session (Post)	9.200	2.059	28.000	4.469	0.0001***
Condition (Sham)	2.200	3.669	38.112	0.600	0.552
Session (Post): Condition (Sham)	−4.067	2.911	28.000	−1.397	0.173

Random effect	Variance	Std. Dev.			
Subject (Intercept)	69.15	8.316			
Residual	31.79	5.638			

***p < 0.0001, **p < 0.001, *p < 0.05.

**Figure 5:**
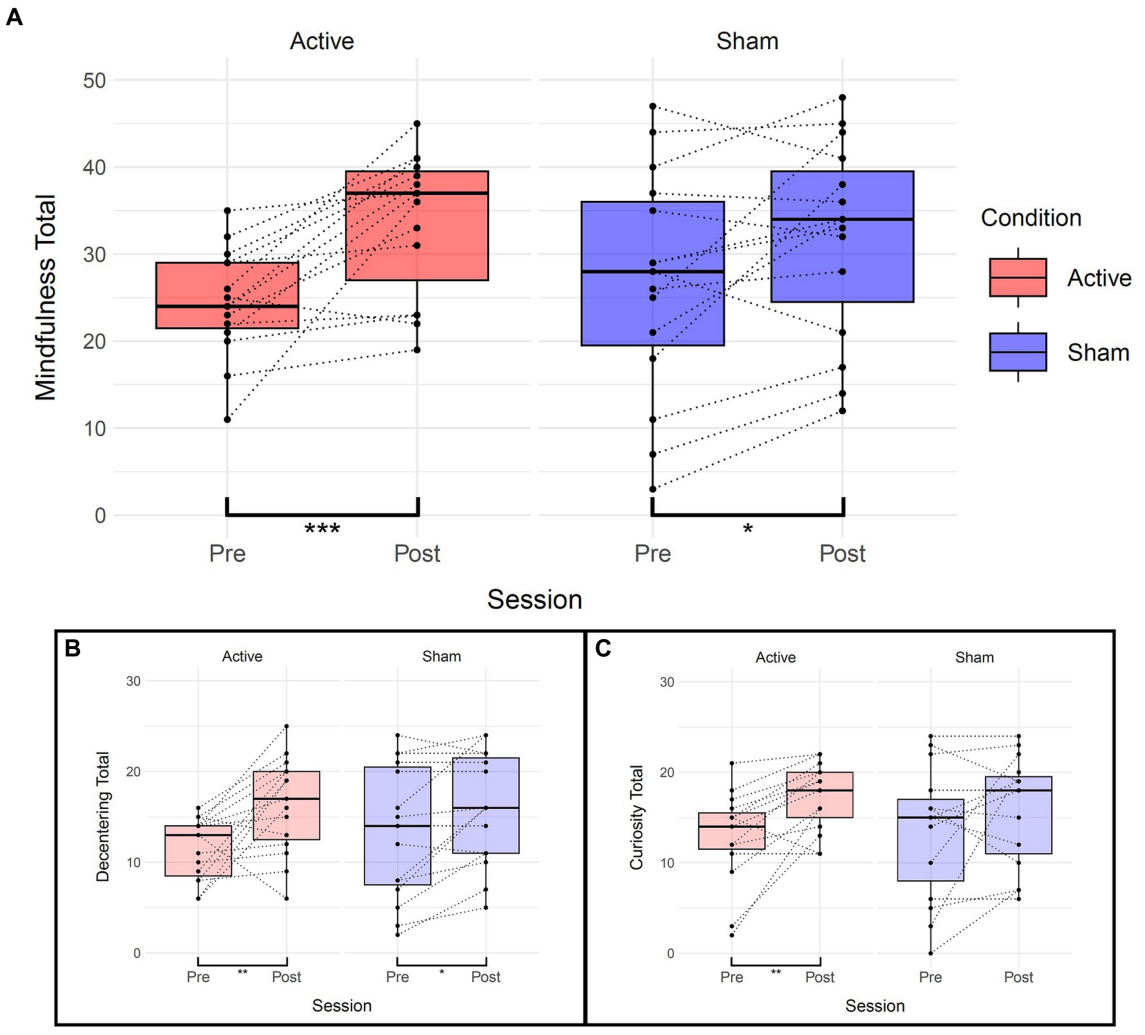
Effects of TFUS on Toronto Mindfulness Scale. Boxplots depicting changes measured by the Toronto Mindfulness Scale. **(A)** Changes in total mindfulness score between pre and post for each active and sham condition. **(B)** Changes in the “Decentering” subscale. **(C)** Changes in the “Curiosity” subscale. ***p < 0.001, **p < 0.01, *p < 0.05.

Paired Wilcoxon rank-sum tests were performed to assess within-session differences for each condition and subscales (“curiosity” and “decentering”) of the Toronto Mindfulness Scale (see Table 5 and Figure 5). Significant differences were found within the active condition between pre and post sessions for the total mindfulness score (t(14) = −4.51, *p* = 0.0004), the “curiosity” subscale (t(14) = −3.98, *p* = 0.001), and the “decentering” subscale (t(14) = −3.24, *p* = 0.006). Increases in the “curiosity” subscale suggest that participants became more open to novelty and more interested in their internal experiences, with less judgment towards them. Increases in the “decentering” subscale reflect an improved ability to be detached towards one’s thoughts and feelings, avoiding identifying with them or perceiving them as accurate reflections of reality (Lau et al., 2006).

**Table 5:** Paired Wilcoxon rank-sum tests for Toronto Mindfulness Scale.

Condition	Comparison	*t* value	df	*p*-value
Active	Pre vs. Post (Total)	−4.509	14	0.0004***
Sham	Pre vs. Post (Total)	−2.472	14	0.027*
Active	Pre vs. Post (Curiosity)	−3.979	14	0.001**
Sham	Pre vs. Post (Curiosity)	−1.590	14	0.134
Active	Pre vs. Post (Decentering)	−3.242	14	0.006**
Sham	Pre vs. Post (Decentering)	−2.870	14	0.012*

***p < 0.0001, **p < 0.01, *p < 0.05.

In the sham condition, significant differences were observed for the total mindfulness score (t(14) = −2.47, *p* = 0.027) and the “decentering” subscale (t(14) = −2.87, *p* = 0.012) but not the “curiosity” subscale (t(14) = −1.59, *p* = 0.134). The linear mixed-effects model did not show significant main effects or interactions overall, but the paired Wilcoxon rank-sum tests consistently indicated significant differences within the TFUS group and inconsistently or of smaller magnitude within the sham group.

##### 3.2.1.2 Visual Analog Mood Scale

We estimated a linear mixed model that included session and condition as fixed effects, their interaction, and a random intercept for subjects, for each score Global Affect and Global Vigor from the VAMS. The formula for the model is [GA or GV] ~ Session * Condition + (1 | Subject) (see Table 6). There was a significant decrease in GV from the baseline to the post-session in the active condition (Estimate = −9.500, SE = 3.686, t(28) = −2.578, *p* = 0.016), with no significant differences in GV observed between conditions at baseline (Estimate = −3.500, SE = 5.563, t(42.591) = −0.629, *p* = 0.533). The interaction between session and condition was not significant (Estimate = 2.500, SE = 5.212, t(28) = 0.480, *p* = 0.635). The random effects structure showed a subject-specific intercept variance of 130.2 (SD = 11.41) and a residual variance of 101.9 (SD = 10.09). For GA, there were no significant differences between sessions in the active condition (Estimate = 2.500, SE = 2.589, t(28) = 0.966, *p* = 0.342), with no significant differences in GA observed between conditions at baseline (Estimate = −4.167, SE = 4.420, t(39.116) = −0.943, *p* = 0.352). The interaction between session and condition was not significant (Estimate = 3.000, SE = 3.661, t(28) = 0.819, *p* = 0.419). The random effects structure showed a subject-specific intercept variance of 96.28 (SD = 9.812) and a residual variance of 50.27 (SD = 7.090).

**Table 6:** Effects estimates for the linear mixed model for Visual Analog Mood Scale.

Global affect					
Fixed effect	Estimate	Std. Error	df	*t* value	*p*-value
(Intercept)	75.000	3.126	39.116	23.995	<10^−16^***
Session (Post)	2.500	2.589	28.000	0.966	0.342
Condition (Sham)	−4.167	4.420	39.116	−0.943	0.352
Session (Post): Condition (Sham)	3.000	3.661	28.000	0.819	0.419

Random effect	Variance	Std. Dev.			
Subject (Intercept)	96.28	9.812			
Residual	50.27	7.090			

Global Vigor					
Fixed effect	Estimate	Std. Error	df	*t* value	*p*-value
(Intercept)	74.167	6.934	42.591	18.854	<10^−16^***
Session (Post)	−9.500	3.686	28.000	−2.578	0.016*
Condition (Sham)	−3.500	5.563	42.591	−0.629	0.533
Session (Post): Condition (Sham)	2.500	5.212	28.000	0.480	0.635

Random effect	Variance	Std. Dev.			
Subject (Intercept)	130.2	11.41			
Residual	101.9	10.09			

***p < 0.001, **p < 0.001, *p < 0.05.

**Figure 6:**
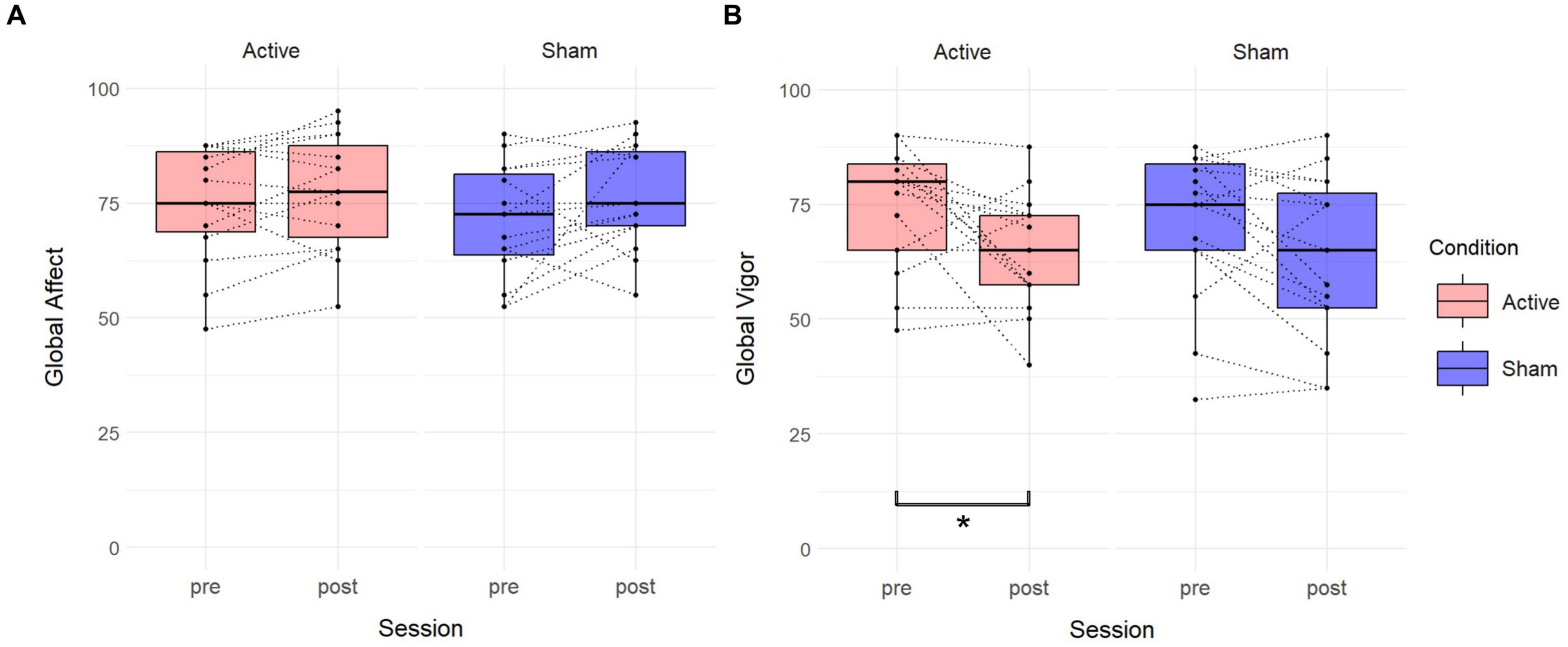
Effects of TFUS on Visual Analog Mood Scale. Boxplots depicting changes measured by the Visual Analog Mood Scale. **(A)** Changes in Global Affect score between pre and post for each active and sham condition. **(B)** Changes in the Global Vigor score between pre and post for each active and sham condition. A significant difference was found in the active condition. *p < 0.05.

Wilcoxon rank-sum tests a significant difference in GV between sessions in the active condition (V = 75, *p* = 0.04238), while no significant differences were observed in GA (V = 32, *p* = 0.2053). In the sham condition, there were no significant differences between sessions in GV (V = 94, *p* = 0.05653), or GA (V = 26, *p* = 0.1015). See Figure 6.

##### 3.2.1.3 Self Scale

**Figure 7:**
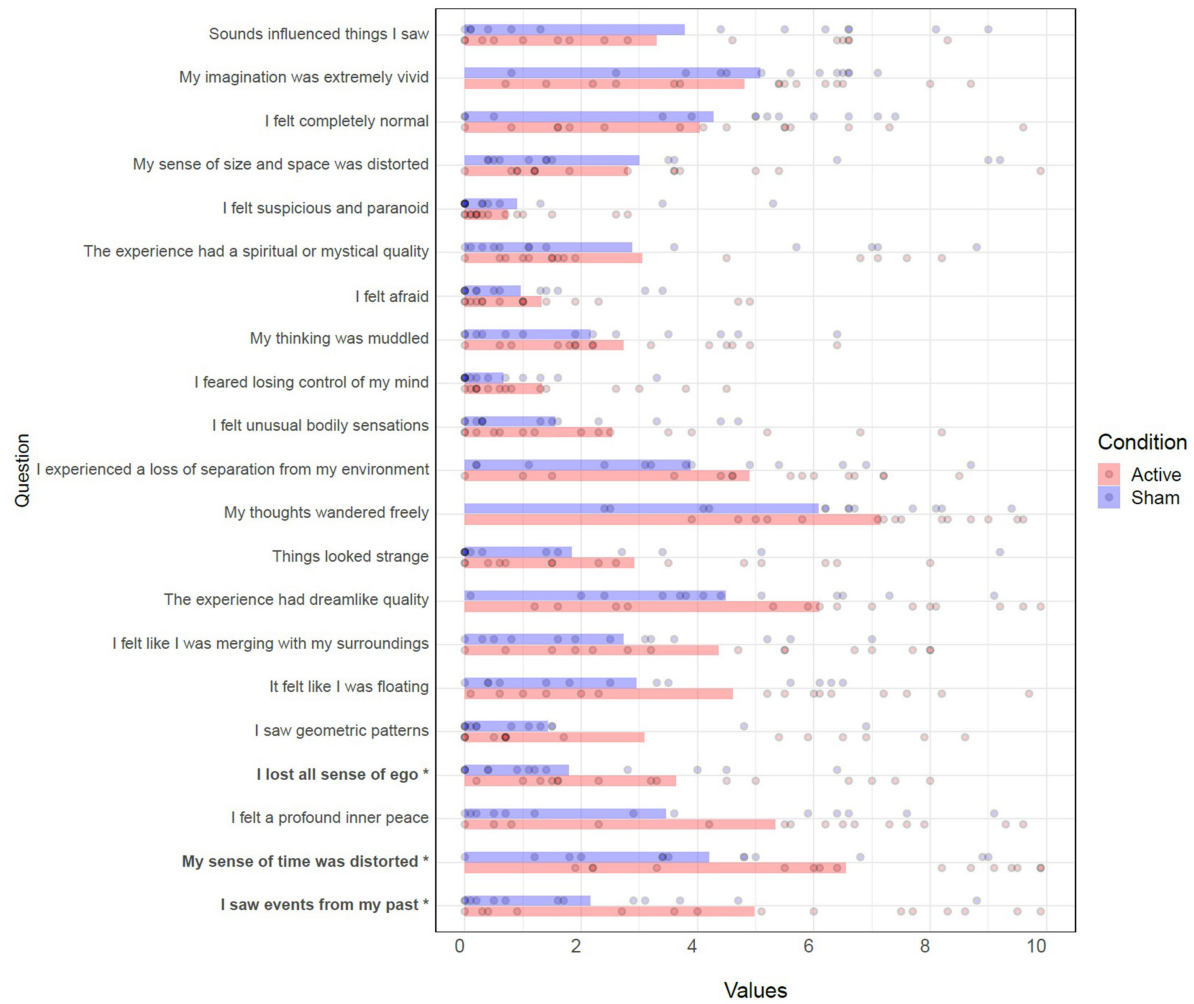
Effects of TFUS on Self Scale. Individual item responses to the Self Scale. *p < 0.05.

Wilcoxon rank-sum tests were performed for each item to compare conditions. Three significant differences (higher scores in active) were found in the items “I saw events from my past” (W = 142, *p* = 0.043), “My sense of time was distorted” (W = 142, *p* = 0.043), and “I lost all sense of ego” (W = 148.5, *p* = 0.020). See Table 7 and Figure 7.

**Table 7:** Wilcoxon rank-sum tests of Self Scale.

Item	Mean difference	Wilcoxon *W*	Wilcoxon *p*
I saw events from my past	2.81	142	0.043*
My sense of time was distorted	2.35	142	0.043*
I felt a profound inner peace	1.89	129.5	0.147
I lost all sense of ego	1.86	148.5	0.020*
I saw geometric patterns	1.66	120	0.308
It felt like I was floating	1.66	127	0.181
I felt like I was merging with my surroundings	1.64	130	0.140
The experience had dreamlike quality	1.61	130.5	0.134
Things looked strange	1.08	129.5	0.144
My thoughts wandered freely	1.07	127.5	0.174
I experienced a loss of separation from my environment	1.02	123	0.249
I felt unusual bodily sensations	0.97	118	0.356
I feared losing control of my mind	0.66	132	0.115
My thinking was muddled	0.57	115.5	0.419
I felt afraid	0.37	114	0.458
The experience had a spiritual or mystical quality	0.18	115.5	0.420
I felt suspicious and paranoid	−0.16	114.5	0.441
My sense of size and space was distorted	−0.20	105	0.747
I felt completely normal	−0.23	92.5	0.836
My imagination was extremely vivid	−0.29	84.5	0.564
Sounds influenced things I saw	−0.48	94	0.890

Items ordered by difference of mean values between active and sham conditions. *p < 0.05.

##### 3.2.1.4 Post experiment questions

No negative experiences or adverse events were reported. When asked to guess which condition they were in, 11/15 of participants in the active condition guessed “stimulation,” and 3/15 of those in the sham condition guessed “stimulation,” Χ^2^ (3, N = 30) = 8.571, *p* = 0.0356. When asked if their mental state changed, 10/15 in the active condition said yes, and 5/15 in the sham condition said yes, Χ^2^ (3, N = 30) = 3.333, *p* = 0.3430. When asked if there were any changes in their “inner talk- or thinking-space” descriptions of felt effects included “made less thoughts,” “better,” “drifted more,” “more calm,” “mind wandering less,” “more active lighter thoughts,” “less effort to think,” “less dark thoughts,” “more calm,” “somewhat more relaxed” in the active group, and “more fluid than usual,” “I was able to organize my thoughts for the day,” “talked more,” “smoother,” “calmed down” in the sham condition. Only 3/30 of participants in either condition said they heard sounds from the transducer, and 2 of those 3 reported that sound as the hum of the amplifier. The other 1 that reported a sound described it as a “buzzing” which may have referred to the actual sound of the transducer emitting at a subaudible PRF of 10.526 Hz.

## 4 Discussion

This study targeted the PCC with TFUS with the aim to reduce resting state functional connectivity in the DMN, predicting that this would result in phenomenological effects on mindfulness and self-referential processing. In an ROI-to-ROI test across the whole brain, the active TFUS group showed reductions in functional connectivity along the midline of the DMN. Additionally, we found that the active TFUS group showed multiple phenomenological changes, namely, an increase in state mindfulness as measured by the Toronto Mindfulness Scale, a reduction in Global Vigor as measured by the Visual Analog Mood Scale, and changes in items related to the sense of ego, sense of time, and seeing memories from the past, as measured by the Self Scale.

We used linear mixed models to estimate a contrast between active and sham TFUS groups, and we did not find significant differences. Similarly, linear mixed models on the phenomenological measurements also did not yield significant effects. This is likely due, in part, to low statistical power for between-subject comparisons with the small sample size and to relatively low ultrasound power.

Our parameters yielded an I_SPTA_ of 293 (mW/cm^2^) and an I_SPPA_ of 5.58 (W/cm^2^) as measured in water. Other studies have used higher intensities on human subjects: Ai et al. (2018) had an Ispta of 6.102 W/cm^2^ and an I_SPPA_ of 16.95 W/cm^2^; Legon et al. (2018) had an I_SPTA_ of 6.192 W/cm^2^ and an I_SPPA_ of 17.2 W/cm^2^. Numerous animal studies have used even higher intensities to elicit their detected effects (Yoo et al., 2011; Kim et al., 2014, 2022; Yoon et al., 2019). Additionally, transmitting ultrasound through the posterior parietal portion of the skull is likely to significantly attenuate the amount of energy that gets transmitted (Mueller et al., 2017). Compared to Sanguinetti et al. (2020), who used the thin “temporal window” for transmission, we transmitted less energy to a deeper neural region. It is likely that higher intensities are needed for more consistent effects.

No significant effects were found in BOLD signal or pc-ASL analysis. This is most likely due to the low intensity of the ultrasound. It may be the case that the intensities used in this study represent the “floor” at which any effects are exhibited in brain activity. That such effects show up in BOLD functional connectivity but not simple BOLD signal suggests more of a tissue-level disruptive or suppressive effect than a straightforward inhibitory effect.

Given that we saw significant increases in mindfulness in the sham group, it is possible that the combination of the MRI scanner, the simulated brain stimulation, and the cues provided by the Toronto Mindfulness Scale and VAMS scales caused subjects to become more present-centered and mindful in their attention. Despite that, the effect sizes in the Toronto Mindfulness Scale changes for the active group were consistently larger compared to the sham group, suggesting TFUS to the DMN may enhance state mindfulness. Future studies that employ increased TFUS power, better targeting methods (such as functional-based targeting), or that combine TFUS with mindfulness training may find a clearer link between DMN changes and state mindfulness.

This study was intended to be a proof-of-concept that it is possible to modulate DMN with focused TFUS. The intention was to make the parameters suppressive by using a low duty cycle. We targeted along the midline, aiming to hit both hemispheres of the PCC. DMN functional connectivity decreased, suggesting a disruption to the stability of the network, including left and right hemisphere locations along the midline decoupling from each other. Although we targeted ventral PCC, other areas like the dorsal PCC and precuneus may have also been in the path of the beam, which may have only added to the meditation-like effects that were observed (Garrison et al., 2015). Given that our tank measurements suggest the beam’s focus was shortened by the skull (see Figure 2 and Table 2), it is very possible that the precuneus was also affected.

Exploratory analysis of each individual time point revealed that the effects appeared more focused to the midline DMN in t_1_ and then became more diffuse, spreading to more distal areas in t_2_. This could indicate the path by which the offline effects dispersed throughout functional networks. In the sham condition, broad reductions were seen in somatomotor and dorsal attention networks in t_1_, but these did not persist into t_2_, and may be the result of a disengagement of attention with external environment and a switch to a more internal mode of processing. These exploratory results should be interpreted cautiously given the relaxed statistical threshold.

It is also worth noting that these effects persisted for at least 30 min (25 min + 6 min scan) after the application of TFUS. Sanguinetti et al. (2020) found offline increases in mood for at least 30 min following TFUS to the right dlPFC, Kim et al. (2023) found offline changes in resting state functional connectivity lasting more than an hour following thalamic stimulation, and Verhagen et al. (2019) found offline effects persisted for more than an hour in macaques. These long-lasting offline effects may be due to glutamate release via the opening of TRPA1 channels in astrocytes (Oh et al., 2019), opening up the possibility of long-term plasticity effects. The full temporal extent of these offline effects should be the subject of future research. However, in the post-experiment questions, participants, even those reporting subjective effects, reported they were back to baseline by the post-questioning (~1 h post sonication).

We did not use masking or sham sound for the transducer because our PRF was in the subaudible range, and PRF is what is heard (Braun et al., 2020). Only one participant reported a “buzzing” that could conceivably be that sound. This, combined with the 11/15 of participants that correctly guessed they were in the active condition (compared to 3/15 for sham) suggests that the distinct subjective experiences played a role in their judgment.

The phenomenological effects of this treatment correspond with what one might predict would result in DMN disruption via the PCC (Brewer et al., 2013), and it demonstrates that TFUS has the potential to be used as an unprecedented neuromodulatory probe for deep brain sites. The brief subjective descriptions of “lighter” or “less” thoughts, “drifting,” and “less effort” correspond with Brewer et al.’s (2013) description of the PCC being involved in “getting caught up in” one’s thoughts. Given that we were able to reduce DMN functional connectivity in naïve undergraduate students, it would be fruitful to measure the same effects on experienced meditators. Individuals who have developed a richer interior clarity and are more capable of manipulating at will their DMN activity might find an added benefit to meditation when TFUS is applied to their PCC, such as was found in PCC reduction neurofeedback studies with experienced meditators (Garrison et al., 2013; van Lutterveld et al., 2017).

We also found reductions in the sense of ego, though to a much lesser degree than what is seen in psychedelics (Lebedev et al., 2015). Our results were also not accompanied by sensory distortions or hallucinations, likely due to the fact the effects of TFUS were isolated to the DMN. This further demonstrates how the spatial specificity of TFUS can be used by researchers to generate causal models about functional brain networks.

These effects also suggest that TFUS shows promise as a therapeutic tool for disorders associated with DMN activity, such as depression and anxiety (Scalabrini et al., 2020). It may serve in other roles, too, given that it provides superior targeting abilities compared to other non-invasive brain stimulation techniques. The offline effects would allow for the TFUS to be embedded in a larger therapeutic intervention that corrects functional imbalances in the brain’s activity.

It should finally be noted that all of this was achieved with a relatively rudimentary targeting approach. Although individual MR-guided neuronavigation was used, the transducer was held by hand, the target was selected by anatomical structure alone, and no efforts were made to minimize skull aberrations. Future research can improve on all these circumstances with robot-controlled transducers, functional-based targeting, and skull aberration modeling. That we saw such significant effects with this approach is extremely promising for the future of TFUS.

## 5 Conclusion

This pilot study showed that TFUS targeted at the PCC can disrupt DMN activity and cause mindfulness-increasing subjective effects. Given these effects, TFUS may serve as a therapeutic tool for treating network dysfunction. Future research should replicate these effects with a larger sample size, more precise targeting methods, and TFUS intensities matching previous human and animal studies. Future research may also investigate what ultrasound parameters, targeting, and modeling methods are optimal for neuromodulation.

## Data availability statement

The raw data supporting the conclusions of this article will be made available by the authors, without undue reservation.

## Ethics statement

The studies involving humans were approved by University of Arizona Human Subjects Protection Program. The studies were conducted in accordance with the local legislation and institutional requirements. The participants provided their written informed consent to participate in this study.

## Author contributions

BL: Data curation, Formal analysis, Investigation, Software, Validation, Visualization, Writing – original draft, Writing – review & editing. JLS: Conceptualization, Formal analysis, Funding acquisition, Investigation, Methodology, Project administration, Software, Supervision, Writing – review & editing. LR: Investigation, Project administration, Writing – review & editing. VM: Conceptualization, Methodology, Supervision, Writing – review & editing. JS: Conceptualization, Methodology, Supervision, Writing – review & editing. SY: Conceptualization, Methodology, Supervision, Writing – review & editing. MF: Formal analysis, Investigation, Software, Writing – review & editing. JJBA: Conceptualization, Methodology, Project administration, Supervision, Writing – review & editing.
