# Supplementary material for: Transcranial focused ultrasound to the posterior cingulate cortex modulates default mode network and subjective experience: an fMRI pilot study
Source: Front Hum Neurosci. 2024 Jun 4;18:1392199. doi: 10.3389/fnhum.2024.1392199 (PMC11184145; doi:10.3389/fnhum.2024.1392199)
Supplement: Supplementary file 1 [file Table_1.DOCX]

Refer to Schaefer et al. 2018 for information on parcellation. Their 17-Network, 100-area parcellation was used in this study.

## Table S1

#### ROI-to-ROI Functional Connectivity Changes in Active in t1>baseline

| Analysis Unit | Statistic | p-unc | p-FDR |
| --- | --- | --- | --- |
| Cluster 1 | Mass=420.42 | 0.000480 | 0.038375 |
| sch171.17Networks_RH_ContC_Cingp_1-sch171.17Networks_LH_ContC_Cingp_1 | T(14)=-6.75 | 0.000009 | 0.023014 |
| sch171.17Networks_LH_ContC_Cingp_1-sch171.17Networks_RH_DefaultA_PFCm_1 | T(14)=-5.22 | 0.00013 | 0.101234 |
| sch171.17Networks_RH_ContC_Cingp_1-sch171.17Networks_RH_DefaultA_PFCm_1 | T(14)=-4.54 | 0.000461 | 0.189893 |
| sch171.17Networks_LH_DefaultA_pCunPCC_1-sch171.17Networks_RH_DefaultA_pCunPCC_1 | T(14)=-4.5 | 0.000499 | 0.189893 |
| sch171.17Networks_LH_ContC_Cingp_1-sch171.17Networks_LH_DefaultA_PFCm_1 | T(14)=-4.13 | 0.001023 | 0.211275 |
| sch171.17Networks_RH_DefaultA_pCunPCC_1-sch171.17Networks_LH_ContC_Cingp_1 | T(14)=-3.9 | 0.001589 | 0.212594 |
| sch171.17Networks_RH_DefaultA_PFCm_1-sch171.17Networks_LH_DefaultA_PFCm_1 | T(14)=-3.88 | 0.001655 | 0.21565 |
| sch171.17Networks_RH_ContC_Cingp_1-sch171.17Networks_LH_DefaultA_PFCm_1 | T(14)=-3.86 | 0.00174 | 0.217583 |
| sch171.17Networks_RH_DefaultA_pCunPCC_1-sch171.17Networks_RH_ContC_Cingp_1 | T(14)=-3.75 | 0.002135 | 0.231904 |
| sch171.17Networks_RH_ContC_Cingp_1-sch171.17Networks_LH_DefaultB_PFCd_1 | T(14)=-3.22 | 0.006114 | 0.362434 |
| sch171.17Networks_LH_DefaultA_pCunPCC_1-sch171.17Networks_RH_ContC_Cingp_1 | T(14)=-3.12 | 0.007486 | 0.362521 |

## Table S2

#### ROI-to-ROI Functional Connectivity Changes in Active in t2>baseline

| Analysis Unit | Statistic | p-unc | p-FDR |
| --- | --- | --- | --- |
| Cluster 1 | Mass=1005.63 | 0.000163 | 0.032030 |
| sch171.17Networks_LH_ContC_Cingp_1-sch171.17Networks_RH_ContC_Cingp_1 | T(14)=-5.87 | 0.000041 | 0.071348 |
| sch171.17Networks_LH_SalVentAttnA_ParMed_1-sch171.17Networks_RH_SalVentAttnA_ParMed_1 | T(14)=-5.36 | 0.0001 | 0.078437 |
| sch171.17Networks_RH_DefaultA_pCunPCC_1-sch171.17Networks_LH_ContC_Cingp_1 | T(14)=-4.69 | 0.000346 | 0.121069 |
| sch171.17Networks_LH_DefaultA_pCunPCC_1-sch171.17Networks_LH_ContC_Cingp_1 | T(14)=-4.52 | 0.00048 | 0.151993 |
| sch171.17Networks_LH_DefaultA_pCunPCC_1-sch171.17Networks_RH_ContC_Cingp_1 | T(14)=-4.42 | 0.000579 | 0.151993 |
| sch171.17Networks_RH_DefaultB_PFCd_1-sch171.17Networks_LH_ContC_Cingp_1 | T(14)=-4.3 | 0.000737 | 0.158275 |
| sch171.17Networks_LH_DefaultA_PFCd_1-sch171.17Networks_RH_DefaultA_pCunPCC_1 | T(14)=-4.07 | 0.001159 | 0.158275 |
| sch171.17Networks_RH_DefaultA_pCunPCC_1-sch171.17Networks_RH_ContC_Cingp_1 | T(14)=-3.96 | 0.001429 | 0.163169 |
| sch171.17Networks_LH_DefaultA_pCunPCC_1-sch171.17Networks_RH_DefaultA_pCunPCC_1 | T(14)=-3.79 | 0.001974 | 0.201631 |
| sch171.17Networks_LH_DefaultB_IPL_1-sch171.17Networks_LH_SalVentAttnA_ParMed_1 | T(14)=-3.63 | 0.002736 | 0.204073 |
| sch171.17Networks_RH_DefaultB_PFCd_1-sch171.17Networks_RH_ContC_Cingp_1 | T(14)=-3.62 | 0.002812 | 0.204073 |
| sch171.17Networks_LH_ContC_Cingp_1-sch171.17Networks_LH_SalVentAttnA_ParMed_1 | T(14)=-3.5 | 0.003568 | 0.20627 |
| sch171.17Networks_LH_DefaultA_PFCd_1-sch171.17Networks_LH_SalVentAttnA_ParMed_1 | T(14)=-3.48 | 0.003699 | 0.206736 |
| sch171.17Networks_LH_DefaultA_PFCd_1-sch171.17Networks_RH_SalVentAttnA_ParMed_1 | T(14)=-3.47 | 0.003784 | 0.209 |
| sch171.17Networks_LH_DefaultB_PFCd_1-sch171.17Networks_LH_ContC_Cingp_1 | T(14)=-3.45 | 0.003934 | 0.209 |
| sch171.17Networks_RH_DefaultA_PFCm_1-sch171.17Networks_LH_ContC_Cingp_1 | T(14)=-3.44 | 0.003969 | 0.209 |
| sch171.17Networks_LH_DefaultB_PFCl_1-sch171.17Networks_LH_SalVentAttnA_ParMed_1 | T(14)=-3.36 | 0.004654 | 0.225286 |
| sch171.17Networks_LH_DefaultB_PFCd_1-sch171.17Networks_RH_ContC_Cingp_1 | T(14)=-3.31 | 0.005143 | 0.232919 |
| sch171.17Networks_LH_ContB_PFClv_1-sch171.17Networks_LH_SalVentAttnA_ParMed_1 | T(14)=-3.26 | 0.005661 | 0.247802 |
| sch171.17Networks_LH_DefaultA_PFCd_1-sch171.17Networks_LH_DefaultA_PFCm_1 | T(14)=-3.21 | 0.006233 | 0.260552 |
| sch171.17Networks_LH_LimbicA_TempPole_2-sch171.17Networks_RH_SalVentAttnA_ParMed_1 | T(14)=-3.19 | 0.006578 | 0.260552 |
| sch171.17Networks_LH_DefaultA_PFCd_1-sch171.17Networks_RH_DefaultA_PFCm_1 | T(14)=-3.17 | 0.006791 | 0.262307 |
| sch171.17Networks_LH_LimbicA_TempPole_2-sch171.17Networks_LH_SalVentAttnA_ParMed_1 | T(14)=-3.17 | 0.006879 | 0.263772 |
| sch171.17Networks_LH_DefaultB_PFCl_1-sch171.17Networks_RH_SalVentAttnA_ParMed_1 | T(14)=-3.09 | 0.008006 | 0.280388 |
| sch171.17Networks_RH_ContC_Cingp_1-sch171.17Networks_LH_SalVentAttnA_ParMed_1 | T(14)=-3.08 | 0.008209 | 0.283684 |
| sch171.17Networks_LH_DefaultA_PFCd_1-sch171.17Networks_RH_ContC_Cingp_1 | T(14)=-3.05 | 0.008675 | 0.289553 |
| sch171.17Networks_LH_LimbicA_TempPole_2-sch171.17Networks_RH_DorsAttnA_ParOcc_1 | T(14)=-3.02 | 0.00915 | 0.289553 |
| sch171.17Networks_RH_LimbicB_OFC_1-sch171.17Networks_LH_ContC_Cingp_1 | T(14)=-3.01 | 0.009445 | 0.291852 |
| sch171.17Networks_LH_DefaultA_pCunPCC_1-sch171.17Networks_LH_SalVentAttnA_ParMed_1 | T(14)=-2.92 | 0.01122 | 0.303797 |
| sch171.17Networks_LH_ContC_Cingp_1-sch171.17Networks_RH_SalVentAttnA_ParMed_1 | T(14)=-2.81 | 0.014041 | 0.341466 |
| sch171.17Networks_LH_DefaultA_PFCd_1-sch171.17Networks_LH_DefaultA_pCunPCC_1 | T(14)=-2.75 | 0.015769 | 0.356143 |
| sch171.17Networks_LH_DefaultB_IPL_1-sch171.17Networks_LH_ContC_Cingp_1 | T(14)=-2.74 | 0.015828 | 0.356143 |
| sch171.17Networks_RH_LimbicB_OFC_1-sch171.17Networks_RH_ContC_Cingp_1 | T(14)=-2.71 | 0.016795 | 0.362085 |
| sch171.17Networks_RH_DefaultB_PFCd_1-sch171.17Networks_RH_SalVentAttnA_ParMed_1 | T(14)=-2.7 | 0.017416 | 0.362085 |
| sch171.17Networks_LH_DefaultB_IPL_1-sch171.17Networks_RH_SalVentAttnA_ParMed_1 | T(14)=-2.65 | 0.019071 | 0.375235 |
| sch171.17Networks_LH_DefaultA_PFCm_1-sch171.17Networks_LH_ContC_Cingp_1 | T(14)=-2.56 | 0.022702 | 0.381179 |
| sch171.17Networks_RH_DefaultB_PFCd_1-sch171.17Networks_LH_SalVentAttnA_ParMed_1 | T(14)=-2.49 | 0.026157 | 0.391466 |
| sch171.17Networks_RH_ContC_Cingp_1-sch171.17Networks_RH_SalVentAttnA_ParMed_1 | T(14)=-2.38 | 0.031779 | 0.425857 |
| sch171.17Networks_LH_ContB_PFClv_1-sch171.17Networks_RH_SalVentAttnA_ParMed_1 | T(14)=-2.35 | 0.033702 | 0.429026 |
| sch171.17Networks_RH_ContC_Cingp_1-sch171.17Networks_RH_DorsAttnA_ParOcc_1 | T(14)=-2.25 | 0.041041 | 0.437336 |
| sch171.17Networks_LH_ContC_Cingp_1-sch171.17Networks_RH_DorsAttnA_ParOcc_1 | T(14)=-2.25 | 0.041413 | 0.437336 |
| sch171.17Networks_LH_DefaultB_IPL_1-sch171.17Networks_RH_ContC_Cingp_1 | T(14)=-2.24 | 0.041461 | 0.437336 |
| sch171.17Networks_RH_DefaultA_PFCm_1-sch171.17Networks_RH_ContC_Cingp_1 | T(14)=-2.19 | 0.045789 | 0.444606 |
| sch171.17Networks_LH_DefaultA_PFCd_1-sch171.17Networks_LH_ContC_Cingp_1 | T(14)=-2.16 | 0.048741 | 0.454772 |
| sch171.17Networks_LH_ContB_PFClv_1-sch171.17Networks_RH_ContC_Cingp_1 | T(14)=-2.15 | 0.049307 | 0.455666 |

## Table S3

#### ROI-to-ROI Functional Connectivity Changes in Sham in t1>baseline

| Analysis Unit | Statistic | p-unc | p-FDR |
| --- | --- | --- | --- |
| Cluster 1 | Mass=1908.51 | 0.000087 | 0.015087 |
| sch171.17Networks_RH_SomMotA_1-sch171.17Networks_RH_DorsAttnB_FEF_1 | T(14)=-7.79 | 0.000002 | 0.003073 |
| sch171.17Networks_RH_SomMotA_1-sch171.17Networks_RH_DorsAttnB_PostC_1 | T(14)=-7.15 | 0.000005 | 0.006007 |
| sch171.17Networks_LH_SomMotB_Cent_1-sch171.17Networks_LH_DorsAttnB_PostC_2 | T(14)=-5.97 | 0.000034 | 0.017043 |
| sch171.17Networks_RH_SomMotB_Cent_1-sch171.17Networks_RH_SomMotA_2 | T(14)=-5.68 | 0.000056 | 0.02541 |
| sch171.17Networks_LH_SomMotB_Cent_1-sch171.17Networks_LH_SomMotA_1 | T(14)=-5.26 | 0.00012 | 0.045632 |
| sch171.17Networks_LH_SomMotB_S2_2-sch171.17Networks_LH_SomMotB_Aud_1 | T(14)=-5.04 | 0.000179 | 0.063429 |
| sch171.17Networks_RH_SomMotA_1-sch171.17Networks_RH_SomMotA_2 | T(14)=-4.93 | 0.00022 | 0.067115 |
| sch171.17Networks_LH_SomMotB_Cent_1-sch171.17Networks_LH_DorsAttnB_FEF_1 | T(14)=-4.92 | 0.000224 | 0.067115 |
| sch171.17Networks_LH_SomMotB_Aud_1-sch171.17Networks_RH_SomMotB_Aud_1 | T(14)=-4.75 | 0.000309 | 0.077805 |
| sch171.17Networks_RH_SomMotB_Cent_1-sch171.17Networks_LH_SomMotA_1 | T(14)=-4.70 | 0.000339 | 0.077805 |
| sch171.17Networks_RH_SomMotB_Cent_1-sch171.17Networks_RH_DorsAttnB_FEF_1 | T(14)=-4.70 | 0.000344 | 0.077805 |
| sch171.17Networks_RH_SomMotB_Aud_1-sch171.17Networks_RH_SomMotB_S2_2 | T(14)=-4.69 | 0.000346 | 0.077805 |
| sch171.17Networks_LH_SomMotB_Aud_1-sch171.17Networks_RH_SomMotB_S2_2 | T(14)=-4.63 | 0.000393 | 0.084559 |
| sch171.17Networks_LH_SomMotB_Cent_1-sch171.17Networks_RH_SomMotA_3 | T(14)=-4.52 | 0.000484 | 0.095919 |
| sch171.17Networks_RH_SomMotA_1-sch171.17Networks_RH_SomMotB_Cent_1 | T(14)=-4.48 | 0.000514 | 0.097831 |
| sch171.17Networks_LH_SomMotB_Cent_1-sch171.17Networks_RH_SomMotA_2 | T(14)=-4.39 | 0.000613 | 0.101217 |
| sch171.17Networks_LH_SomMotB_Cent_1-sch171.17Networks_RH_DorsAttnB_PostC_1 | T(14)=-4.33 | 0.000695 | 0.101217 |
| sch171.17Networks_RH_SomMotB_Cent_1-sch171.17Networks_LH_DorsAttnB_PostC_2 | T(14)=-4.25 | 0.000805 | 0.111817 |
| sch171.17Networks_RH_SomMotA_1-sch171.17Networks_LH_DorsAttnB_FEF_1 | T(14)=-4.07 | 0.001151 | 0.13348 |
| sch171.17Networks_LH_SomMotB_Cent_1-sch171.17Networks_RH_DorsAttnB_FEF_1 | T(14)=-4.00 | 0.001305 | 0.144552 |
| sch171.17Networks_RH_SomMotA_1-sch171.17Networks_RH_ContA_PFCl_2 | T(14)=-3.99 | 0.001338 | 0.144552 |
| sch171.17Networks_RH_SomMotB_Cent_1-sch171.17Networks_RH_SomMotA_3 | T(14)=-3.98 | 0.001367 | 0.144552 |
| sch171.17Networks_RH_SomMotB_S2_2-sch171.17Networks_RH_DorsAttnB_PostC_1 | T(14)=-3.98 | 0.001377 | 0.144552 |
| sch171.17Networks_RH_SomMotB_Cent_1-sch171.17Networks_RH_DorsAttnB_PostC_1 | T(14)=-3.95 | 0.001444 | 0.144552 |
| sch171.17Networks_LH_SomMotB_Cent_1-sch171.17Networks_LH_DorsAttnB_PostC_1 | T(14)=-3.95 | 0.001449 | 0.144552 |
| sch171.17Networks_RH_SomMotB_Cent_1-sch171.17Networks_LH_DorsAttnB_FEF_1 | T(14)=-3.89 | 0.00163 | 0.154114 |
| sch171.17Networks_RH_SomMotB_S2_1-sch171.17Networks_RH_SomMotB_S2_2 | T(14)=-3.80 | 0.001935 | 0.162322 |
| sch171.17Networks_RH_SomMotA_1-sch171.17Networks_LH_DorsAttnB_PostC_2 | T(14)=-3.74 | 0.0022 | 0.175611 |
| sch171.17Networks_LH_SomMotB_S2_2-sch171.17Networks_RH_SomMotB_S2_1 | T(14)=-3.41 | 0.00419 | 0.265895 |
| sch171.17Networks_RH_SomMotA_1-sch171.17Networks_LH_SomMotA_1 | T(14)=-3.38 | 0.004453 | 0.272139 |
| sch171.17Networks_LH_SomMotB_Cent_1-sch171.17Networks_LH_DorsAttnA_TempOcc_1 | T(14)=-3.33 | 0.004914 | 0.28508 |
| sch171.17Networks_LH_SomMotB_S2_2-sch171.17Networks_RH_SomMotB_Aud_1 | T(14)=-3.33 | 0.004934 | 0.28508 |
| sch171.17Networks_RH_SomMotB_S2_2-sch171.17Networks_RH_DorsAttnB_FEF_1 | T(14)=-3.33 | 0.004953 | 0.28508 |
| sch171.17Networks_LH_SomMotB_S2_2-sch171.17Networks_RH_SomMotB_S2_2 | T(14)=-3.24 | 0.005959 | 0.29795 |
| sch171.17Networks_RH_SomMotB_Cent_1-sch171.17Networks_RH_ContA_PFCl_2 | T(14)=-3.17 | 0.006779 | 0.311487 |
| sch171.17Networks_LH_SomMotA_1-sch171.17Networks_RH_DorsAttnB_FEF_1 | T(14)=-3.16 | 0.006939 | 0.31247 |
| sch171.17Networks_RH_SomMotB_Cent_1-sch171.17Networks_RH_DorsAttnA_TempOcc_1 | T(14)=-3.10 | 0.007873 | 0.324731 |
| sch171.17Networks_RH_SomMotB_Cent_1-sch171.17Networks_LH_DorsAttnA_TempOcc_1 | T(14)=-3.09 | 0.007944 | 0.324731 |
| sch171.17Networks_LH_SomMotB_Cent_1-sch171.17Networks_LH_ContA_PFCl_2 | T(14)=-3.00 | 0.009558 | 0.355846 |
| sch171.17Networks_LH_SomMotA_1-sch171.17Networks_LH_DorsAttnB_FEF_1 | T(14)=-2.96 | 0.010415 | 0.361181 |
| sch171.17Networks_RH_SomMotA_1-sch171.17Networks_RH_SomMotA_3 | T(14)=-2.90 | 0.011648 | 0.386958 |
| sch171.17Networks_RH_SomMotB_Cent_1-sch171.17Networks_LH_ContA_PFCl_2 | T(14)=-2.86 | 0.012514 | 0.394544 |
| sch171.17Networks_LH_SomMotA_1-sch171.17Networks_RH_SomMotA_2 | T(14)=-2.81 | 0.013879 | 0.404367 |
| sch171.17Networks_RH_SomMotB_Cent_1-sch171.17Networks_LH_DorsAttnB_PostC_1 | T(14)=-2.77 | 0.014942 | 0.408635 |
| sch171.17Networks_RH_SomMotB_S2_2-sch171.17Networks_RH_SomMotA_1 | T(14)=-2.76 | 0.01536 | 0.410292 |
| sch171.17Networks_LH_SomMotB_Cent_1-sch171.17Networks_LH_DorsAttnA_ParOcc_1 | T(14)=-2.75 | 0.015754 | 0.410292 |
| sch171.17Networks_LH_SomMotB_S2_2-sch171.17Networks_LH_SomMotB_S2_1 | T(14)=-2.74 | 0.015801 | 0.410292 |
| sch171.17Networks_RH_SomMotA_1-sch171.17Networks_RH_DorsAttnA_TempOcc_1 | T(14)=-2.67 | 0.018362 | 0.426933 |
| sch171.17Networks_LH_SomMotB_Cent_1-sch171.17Networks_LH_DorsAttnB_PostC_3 | T(14)=-2.61 | 0.020441 | 0.437962 |
| sch171.17Networks_RH_SomMotA_1-sch171.17Networks_LH_DorsAttnB_PostC_1 | T(14)=-2.61 | 0.020634 | 0.437962 |
| sch171.17Networks_RH_SomMotB_S2_1-sch171.17Networks_RH_SomMotB_Aud_1 | T(14)=-2.60 | 0.020911 | 0.437962 |
| sch171.17Networks_LH_SomMotB_Cent_1-sch171.17Networks_RH_SomMotA_4 | T(14)=-2.60 | 0.021146 | 0.437962 |
| sch171.17Networks_RH_SomMotB_Cent_1-sch171.17Networks_LH_SomMotB_Cent_1 | T(14)=-2.59 | 0.021323 | 0.439793 |
| sch171.17Networks_LH_SomMotA_1-sch171.17Networks_RH_SomMotA_3 | T(14)=-2.55 | 0.02298 | 0.449627 |
| sch171.17Networks_LH_SalVentAttnA_Ins_1-sch171.17Networks_RH_SomMotB_S2_2 | T(14)=-2.54 | 0.023371 | 0.450016 |
| sch171.17Networks_RH_SomMotB_S2_2-sch171.17Networks_RH_SomMotA_2 | T(14)=-2.53 | 0.02424 | 0.450016 |
| sch171.17Networks_RH_SomMotA_1-sch171.17Networks_LH_SomMotB_Cent_1 | T(14)=-2.50 | 0.025374 | 0.457475 |
| sch171.17Networks_LH_SalVentAttnA_Ins_1-sch171.17Networks_LH_SomMotB_Aud_1 | T(14)=-2.45 | 0.02786 | 0.47135 |
| sch171.17Networks_LH_SomMotA_1-sch171.17Networks_RH_DorsAttnB_PostC_1 | T(14)=-2.45 | 0.027995 | 0.47135 |
| sch171.17Networks_RH_SomMotB_S2_2-sch171.17Networks_LH_SomMotA_1 | T(14)=-2.44 | 0.028425 | 0.472322 |
| sch171.17Networks_LH_SomMotB_Cent_1-sch171.17Networks_LH_SomMotA_2 | T(14)=-2.39 | 0.031402 | 0.484383 |
| sch171.17Networks_LH_SomMotB_Cent_1-sch171.17Networks_RH_DorsAttnA_TempOcc_1 | T(14)=-2.37 | 0.032589 | 0.486141 |
| sch171.17Networks_LH_SalVentAttnA_Ins_1-sch171.17Networks_LH_SomMotB_S2_2 | T(14)=-2.35 | 0.033991 | 0.486141 |
| sch171.17Networks_RH_SomMotA_4-sch171.17Networks_RH_DorsAttnB_FEF_1 | T(14)=-2.32 | 0.03611 | 0.496846 |
| sch171.17Networks_LH_SomMotB_S2_1-sch171.17Networks_RH_SomMotB_S2_2 | T(14)=-2.31 | 0.036638 | 0.496846 |
| sch171.17Networks_RH_SomMotA_4-sch171.17Networks_LH_DorsAttnB_FEF_1 | T(14)=-2.29 | 0.038403 | 0.498276 |
| sch171.17Networks_RH_SomMotB_S2_2-sch171.17Networks_RH_ContA_PFCl_2 | T(14)=-2.27 | 0.039267 | 0.503559 |
| sch171.17Networks_RH_SomMotB_S2_2-sch171.17Networks_LH_DorsAttnB_PostC_1 | T(14)=-2.27 | 0.039898 | 0.505494 |
| sch171.17Networks_RH_SomMotA_1-sch171.17Networks_RH_SomMotA_4 | T(14)=-2.15 | 0.049933 | 0.532769 |
